# Emergence of superconductivity in doped H_2_O ice at high pressure

**DOI:** 10.1038/s41598-017-07145-4

**Published:** 2017-07-28

**Authors:** José A. Flores-Livas, Antonio Sanna, Miglė Graužinytė, Arkadiy Davydov, Stefan Goedecker, Miguel A. L. Marques

**Affiliations:** 1 Department of Physics, Universität Basel, Klingelbergstr. 82, 4056 Basel, Switzerland; 2 Max-Planck Institut of Microstructure Physics, Weinberg 2, 06120 Halle, Germany; 3 Institut für Physik, Martin-Luther-Universität Halle-Wittenberg, D-06099 Halle, Germany

## Abstract

We investigate the possibility of achieving high-temperature superconductivity in hydrides under pressure by inducing metallization of otherwise insulating phases through doping, a path previously used to render standard semiconductors superconducting at ambient pressure. Following this idea, we study H_2_O, one of the most abundant and well-studied substances, we identify nitrogen as the most likely and promising substitution/dopant. We show that for realistic levels of doping of a few percent, the phase X of ice becomes superconducting with a critical temperature of about 60 K at 150 GPa. In view of the vast number of hydrides that are strongly covalent bonded, but that remain insulating up to rather large pressures, our results open a series of new possibilities in the quest for novel high-temperature superconductors.

## Introduction

The theoretical prediction^1^ and subsequent experimental discovery^2^ of superconductivity in H_3_S at 200 GPa, with the record critical temperature (T_C_) of 203 K, rekindled the century-old dream of a room temperature superconductor. The mechanism for superconductivity is clearly understood within the strong coupling theory of Bardeen, Cooper, and Schrieffer^3^ and the high T_C_ arises from the strong electron-phonon coupling due to the peculiar electronic structure of this system under pressure^4–9^. The aim of this research effort is to better understand how high critical temperatures can be achieved and if the same mechanisms can work at lower pressures and/or even higher (room temperature) T_C_ in other materials. In this quest for novel high-T_C_ superconductors, many other materials have been proposed. As the presence of hydrogen seems to be fundamental to reach the very high phonon frequencies, strong electron-phonon coupling, and therefore large critical temperatures^10–14^, the major emphasis has been given to other hydrides^5, 15–29^ such as silane^17, 30–33^, disilane^34^, hydrogen sulfide^6–8, 35–41^ hydrogen selenide^9^, phosphine^42, 43^, etc.

Unfortunately, many (if not most) chemical compounds containing hydrogen only metallize at extremely high pressures. The paradigmatic case is pure hydrogen, whose metallic state is the ground-state structure only above 500 GPa^44–49^. There are certainly other phases that are metallic at lower pressure, but these are often thermodynamically unstable, and therefore difficult, if not impossible, to access experimentally.

A possible, but until now overlooked, solution is doping. It is well known that by introducing enough electron- or hole-donating impurities one can render a semiconducting system metallic and even superconducting. This strategy was already successful in inducing superconductivity in diamond (doped with boron) in 2004^50^, silicon (doped with boron^51^), germanium (doped with gallium^52^), and silicon carbide (doped with boron^53^ or aluminum^54^). Transition temperatures are unfortunately rather low, remaining below 4 K.

In this work we follow this strategy, and investigate if the combination of doping and high pressure can be used to obtain high-temperature superconductivity in hydrides. We select as an example one of the most abundant, and also one of the best studied, hydrides, namely H_2_O. Note that undoped H_2_O remains insulating up to the terapascal range of pressures. In fact, its metallization was predicted to occur beyond 5 TPa^55–58^.

## Results

### Covalent phase of ice under pressure

Despite its simple chemical formula, H_2_O appears in nature in all three common states of matter and it has one of the most complex phase diagrams known ref. 59. Over a dozen of different crystallographic phases have been reported or predicted in a wide range of temperatures and pressures^55–58, 60–76^.

At ambient pressure and low temperatures ice assumes^60^ its phase I, where oxygen has four hydrogen neighbors: two covalently bonded (forming the H_2_O molecule) and two connected by hydrogen bonds to neighboring H_2_O molecules. Below 200 K, phase I transforms to phase XI, and under compression to phase IX, stable in the range from 0.1 to 1 GPa. Under further compression, and at very low temperatures, the phase VIII dominates up to 60–80 GPa. This molecular crystal can be seen as an ordered and symmetric version of phase VII that occurs at high temperatures. At 80–90 GPa emerges the cuprite-type ice-X, characterized by static, symmetric O–H bonds^77–79^.

**Figure 1:**
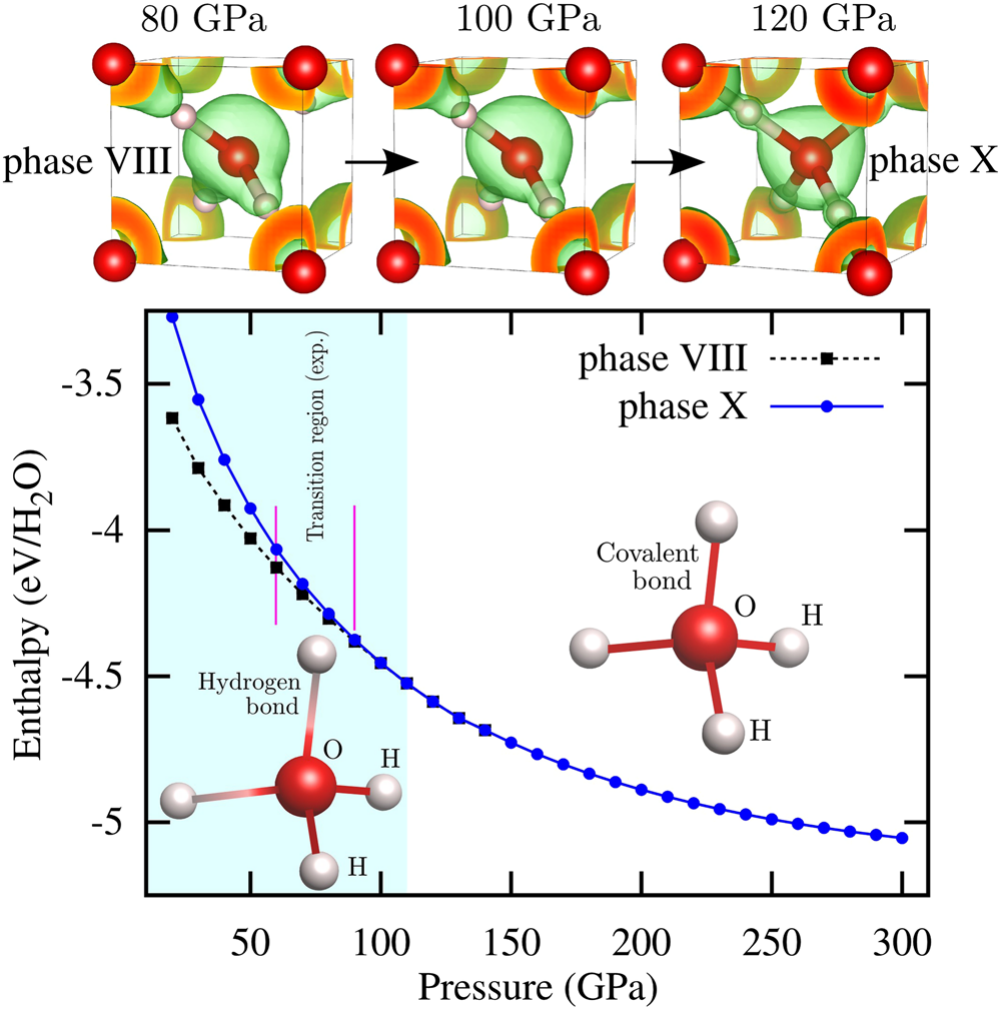
Top sketch: Calculated electron localization function (ELF) at *n* = 0.6 for ice as a function of pressure. Bottom panel: Calculated enthalpy for the VIII and X phases of H_2_O ice. Values are given with respect to the elemental decomposition H_2_ + O. The experimental transition region between phases VIII and X is marked within lines according to Goncharov *et al*.^107^. In our DFT-PBE calculations the transition from a molecular ice-crystal to the fully symmetric covalent phase X is completed at 120 GPa.

Figure 1 shows the calculated enthalpy for two phases of ice. In agreement with the experimental knowledge^78, 79^, we find that at low pressure phase VIII dominates. Above 110 GPa phase VIII undergoes a transition to the proton-symmetric and experimentally confirmed phase X. Benoit *et al*.^80^ have shown that from 102 GPa onwards the proton-ordered symmetric ice X emerges, i.e. localized protons at the bond mid-points. At these pressures, quantum effects do not play such a crucial role as they do for the molecular “antiferroelectric” ice VIII and “paraelectric” ice VII^81^. Phase X is the dominant structure of ice up to 210 GPa^82^. This phase is extremely interesting from our point of view, as it is no longer a molecular crystal and exhibits a complete covalent character, as indicated by the behavior of the electron localization function^83^ (see top panel of Fig. 1). This is absolutely essential for the appearance of doping induced superconductivity, otherwise impurities would just introduce localized states that can not participate in the formation of Cooper pairs.

### Electronic structure of doped ice under pressure

In order to study doping in H_2_O, we created supercells of ice X under pressure for a wide range of doping values H_2_O_1−*x*_ Dopant_*x*_, with *x* = 25%, 12.5%, 6.25%, and 4.16%. Full structural relaxation (volume and lattice vectors) were carried out for the supercells (12 atoms cell for *x* = 25%, 24 atoms cell for 12.5%, 48 atoms cell for 6.25% and 72 atoms cell for 4.16%) at 150 GPa. For low doping (4.16–6.25%), we find fairly small modifications of the crystal structure of ice-X. On the contrary, larger doping levels (25%) induce deformation around the N sites, even if the cuprite-type ice-X global structure is preserved.

**Figure 2:**
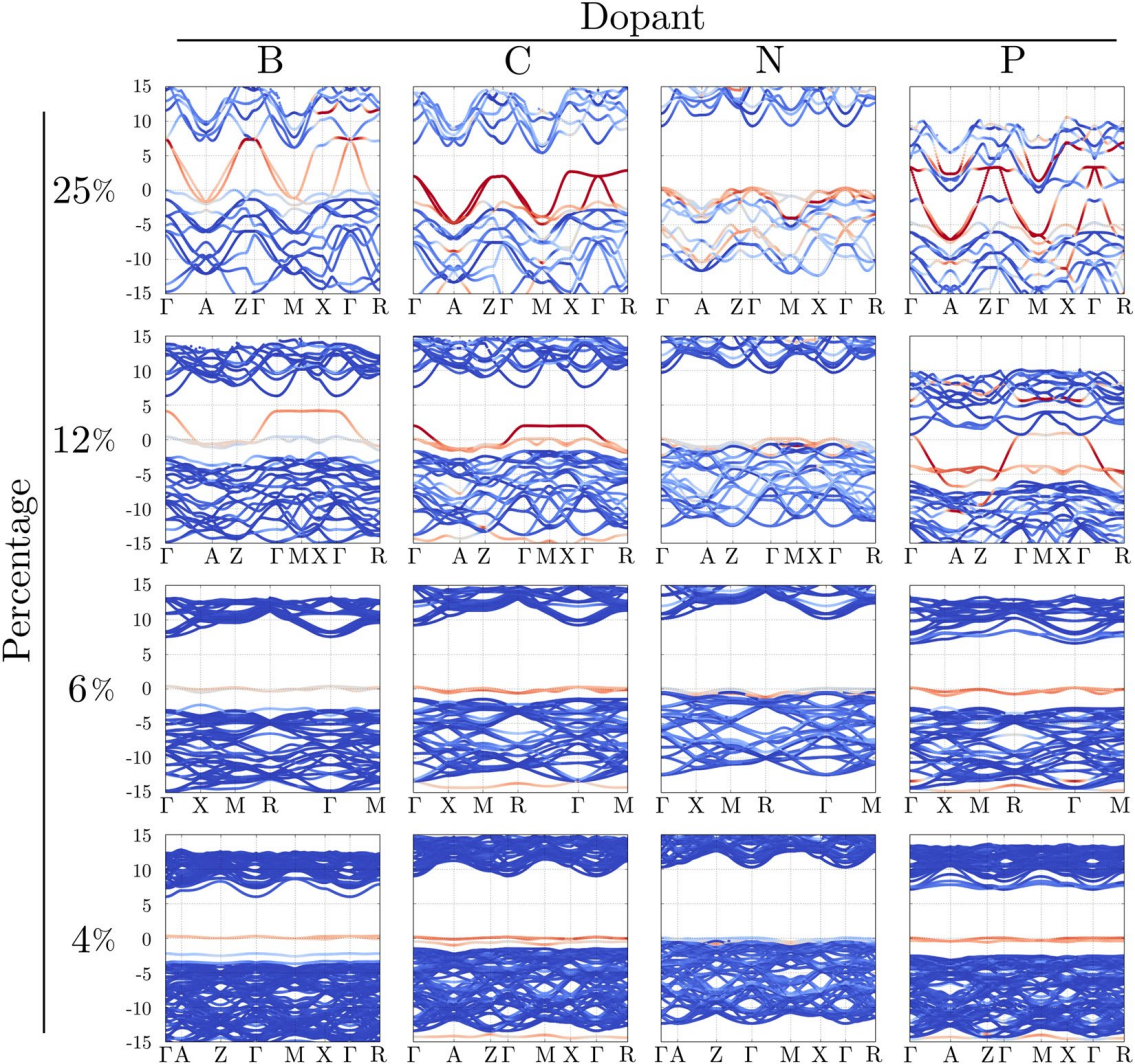
Electronic band structure for different dopants (B, C, N and P) in the phase-X of ice at 150 GPa. The Fermi level is set to 0 eV and the color scale represents the overlap of the Kohn-Sham states on the atomic orbitals of the dopant: red means large overlap (dopant states dominant), gray intermediate, while blue means small (hydrogen and oxygen states dominant).

Figure 2 depicts the electronic band structure obtained when boron, carbon, nitrogen and phosphorous are used to dope ice-X at 150 GPa. In these plots the color scale represents the overlap of the Kohn-Sham states on the atomic orbitals of the dopant: red means large overlap (dopant states dominant), gray intermediate, while blue means small (hydrogen and oxygen states dominant). For large doping with boron and phosphorous (12.5% and 25%), the dispersive band coming from the dopant completely closes the gap in ice-X, while for 4.16% and 6.25% the dopant states form impurity molecular-like bands. These atoms are therefore not suitable to hole-dope ice-X. The case of carbon is intermediate: at low doping we see again the formation of impurity bands, while at higher levels we do see some hybridization between the carbon and the ice bands at the top of the valence. In general we find a higher density of states at the Fermi level with B, C and P acting as a dopant, however the band structure shows mostly localized molecular states which are detrimental to superconductivity. It is important to mention that, since ice-X is highly symmetric (cubic structure, *P* − 43*m*, space group 215) there is only one site to substitute. A study of all possible site/defect substitutions of oxygen by dopants for high doping levels (i.e. 12.5% and 25%) is clearly beyond the scope of this work (and would probably require more advanced techniques such as cluster expansions). However, our results provide a clear general trend of the physics of doped ice under pressure (See Table 1).

**Table 1:** Electron-phonon coupling constant (*λ*), logarithmic average of the phonon frequencies (*ω*_log_), superconducting critical temperature (T_C_) and integral of *α* ^2^ *F*.

	Doping			
	4.16%	6.25%	12.5%	25%
*λ*	0.64	0.85	0.67	0.66
*ω*_log_ (meV)	102	90	84	68
T_C_ (K)	34.4	60.4	33.3	25.6
$\int \,{\alpha }^{2}F$ (meV)	44.2	51.8	40.4	34.8

**Figure 3:**
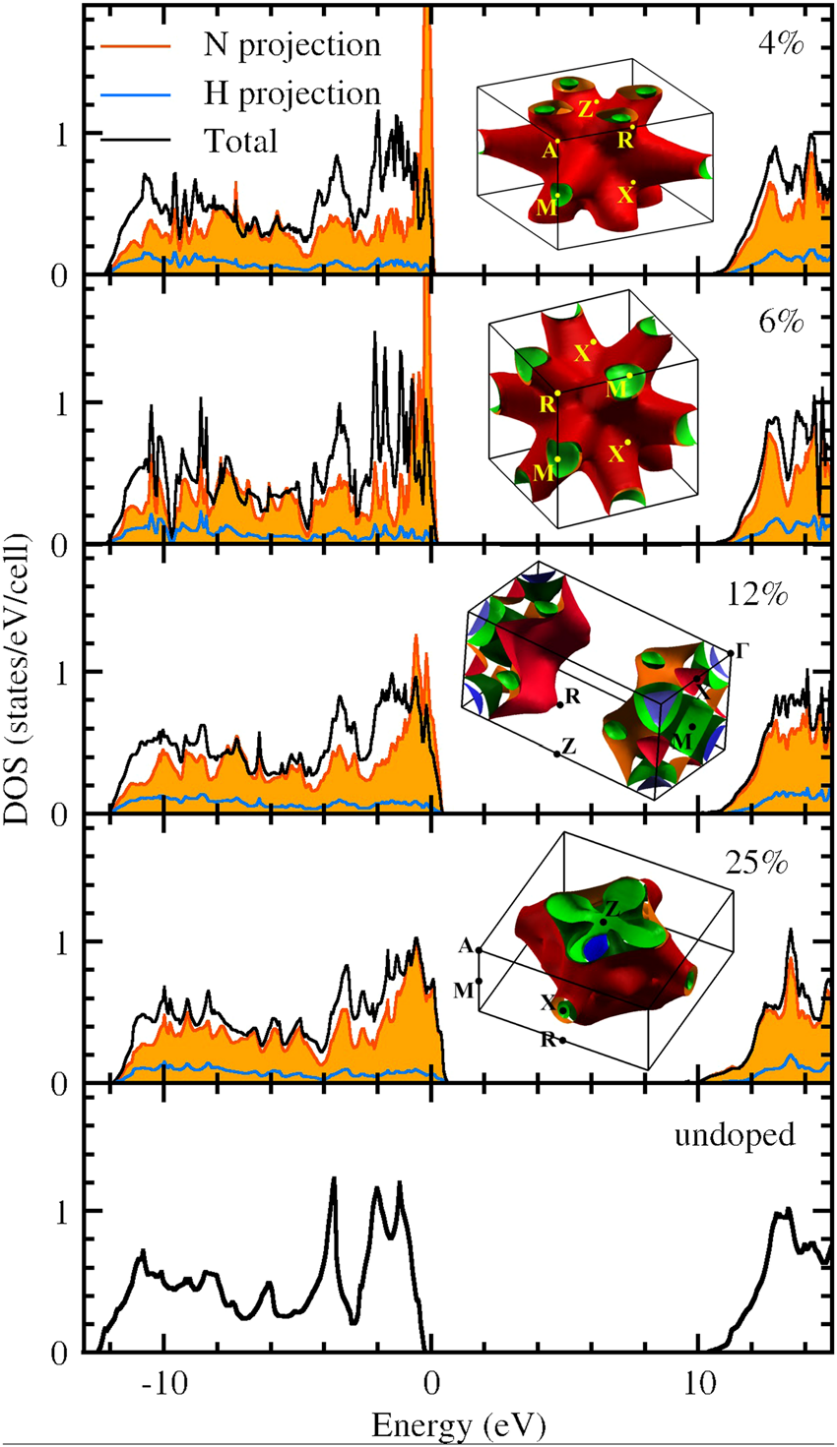
Density of states (DOS) and Fermi surfaces of undoped and N-doped phase X of H_2_O at 150 GPa. Thick black lines are the total DOS, light-blue lines are the H-projected DOS. For plotting convenience these are scaled down by the number of formula units per simulation cell (24, 16, 8 and 4 respectively for the 4.16%, 6.25%, 12.5% and 25% doped systems). Filled orange curves are the projection on nitrogen atomic states (not scaled). All doped systems are metallic featuring one or two small electron pockets around the gamma point (blue/green) an one or two large open surfaces (red/green).

In contrast to the other dopants, nitrogen induces hole doping in ice-X (see Fig. 2) and, as shown in the Supplemental Material of ref. 84 it is also the most likely non iso-valent element able to substitute oxygen. The bottom panel in Fig. 3 shows in detail the electronic structure of ice-X: it is an insulator with a PBE electronic band gap of about 10 eV and does not undergo major modifications at least up to 300 GPa. According to our calculations, the insulator-metal transition is achievable starting from values of 4% nitrogen doping (as seen in Fig. 2). For 25% doping, the top valence is mostly dominated by nitrogen states forming a fairly dispersive band. For values between 4% and 12.5%, oxygen and nitrogen strongly hybridize in the top valence forming the metallic states (gray colors in the plot).

Electronically, it is clear that nitrogen doping introduces holes in the ice X crystal. These hole states are, as intended, hybrid O–N states, as can be seen from the projected density of states in Fig. 3. At high doping (12.5% and 25%) the states at the Fermi level are homogeneous O–N hybrids, meaning that the density of N states is simply proportional to the fraction of N atoms. On the other hand, at lower doping (4.16% and 6.25%) the N projected DOS, although overall smaller, is larger than the N/O fraction and shows a sharp peak close to the Fermi level, indicating that the induced holes are more localized on the N sites. We note that the two last values are realistic and similar to the doping values used to render diamond or silicon superconducting at ambient pressure^85^.

### Stability of doped-ice under pressure

**Figure 4:**
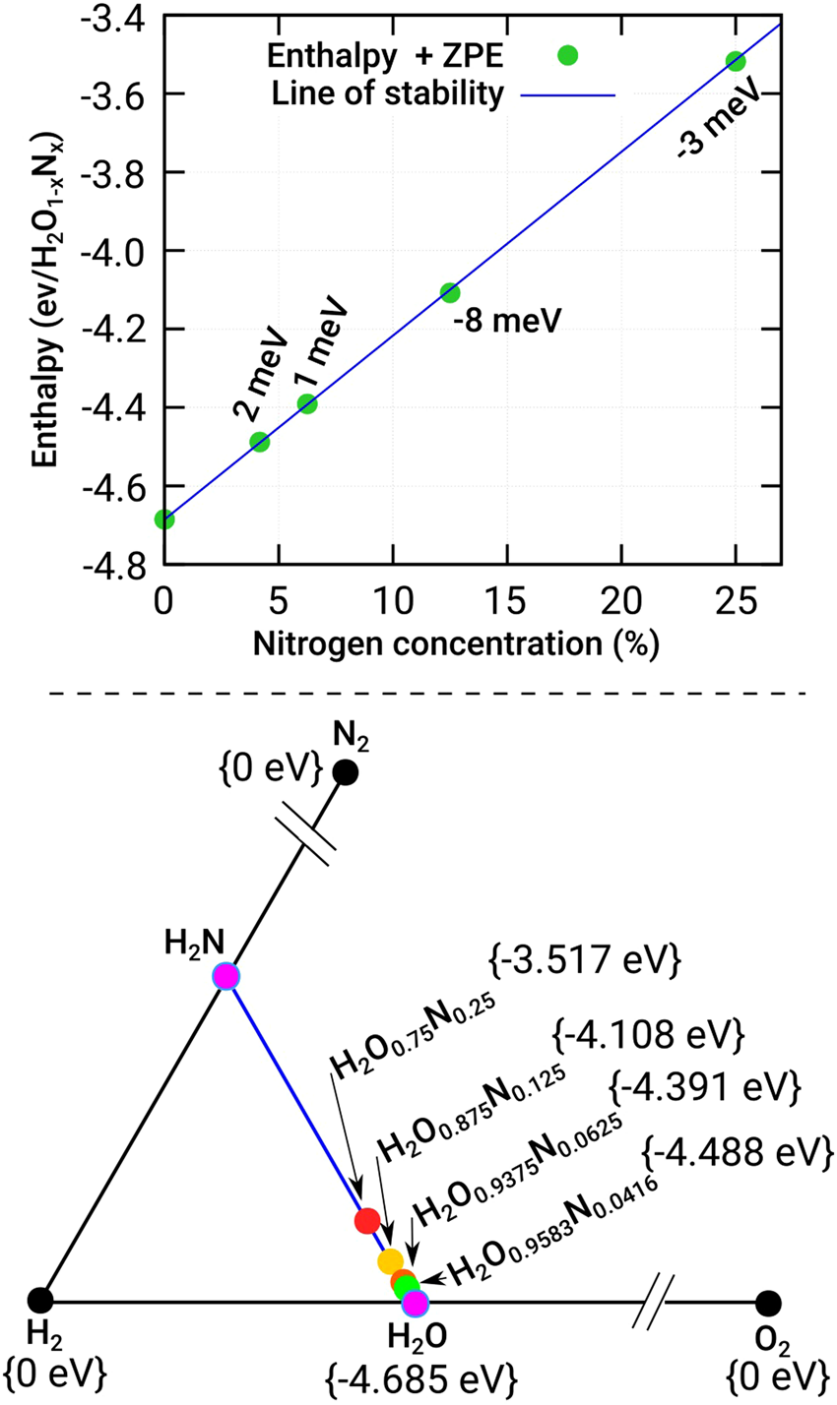
Top panel: calculated formation energy and stability line including zero point energy corrections (ZPE) for ice-X as function of nitrogen content. Bottom panel: part of the ternary phase diagram under consideration in this work, solid black dots represent the ground-state of experimentally know structures of H_2_, O_2_ and N_2_ occurring at 150 GPa.

We studied the stability of the doped compositions with nitrogen by means of total energy DFT calculations. The top panel in Fig. 4 shows the calculated formation energy including zero point energy corrections (ZPE) for ice-X as a function of nitrogen content. The inclusion of ZPE has been show to be fundamental to describe the energetics of hydrogen-based systems, specially at high pressures^86, 87^. Our systems lie on a straight-line drawn between H_2_O and the hypothetical H_2_N system.

The doped structures of ice-X are enthalpically stable towards decomposition to their elemental solids, and all the compositions lie within a mere couple of meV per formula unit above the stability line. The bottom panel zooms in the area of the ternary phase diagram under consideration in this work, solid black dots represent the ground-state enthalpy of experimentally known structures of H_2_, O_2_ and N_2_ occurring at 150 GPa.

**Figure 5:**
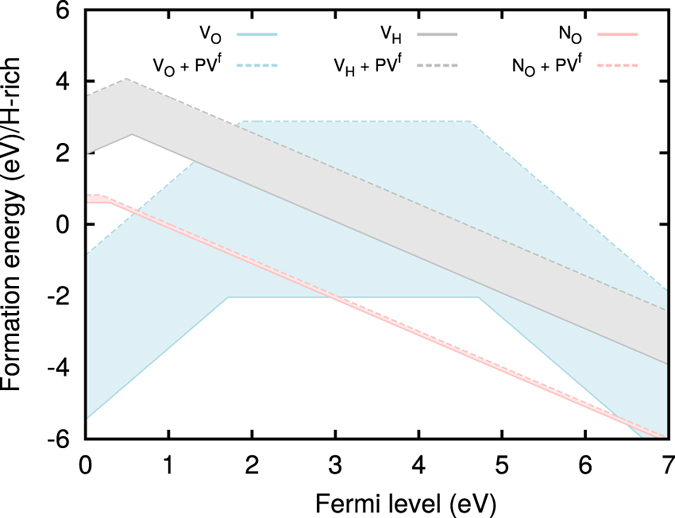
Defect formation energies at *P* = 150 GPa in ice-X as a function of Fermi level for oxygen vacancy *V*_*O*_, hydrogen vacancy *V*_*H*_ and nitrogen on a substitutional O-site *N*_*O*_, under H-rich conditions. Solid lines show the calculated formation energies, while the dashed lines show the formation enthalpies (including the *PV* ^*f*^ contribution).

We can also look at this problem from a different perspective, namely by considering N as a substitutional dopant for an oxygen atom (*N*_*O*_), calculating its formation energy, and comparing it to those of intrinsic defects, such as oxygen (*V*_*O*_) and hydrogen (*V*_*H*_) vacancies. Figure 5 shows the calculated formation energies and enthalpies as a function of the Fermi level inside the band gap for hydrogen-rich experimental conditions. These are the most favorable conditions for *V*_*O*_ and hence *N*_*O*_ formation. The slopes in the formation energy plot indicate the stable charge state of the defect. For Fermi level values ﻿very close to the valence band maximum (relevant for hole conductivity) Fig. 5 indicates that the most likely defect is ${V}_{O}^{+2}$ with ${N}_{O}^{0}$ only 1 eV higher in energy. This is a rather low value, indicating that N-doping is likely to be possible. However, our study also suggests that hole compensation by electrons provided by oxygen vacancies in ice-X cannot be ruled out. The shaded areas outline the large impact that the inclusion of the *PV* ^*f*^ term has on the formation energies at these pressures. Unfortunately, the formation volume *V* ^*f*^ is not technically defined for non-elemental solids and must be approximated (see supplementary material), leading to some ambiguity in the exact values for the formation enthalpies.

### Superconducting properties

There are many possible ways to study theoretically the effect of doping on the superconducting properties. The simplest way is by shifting rigidly the Fermi level, leaving both Kohn-Sham eigenvalues and eigenfunctions unchanged. The resulting phonon spectrum and electron-phonon scattering amplitude can then be used within an Eliashberg^88, 89^ scheme to compute the superconducting critical temperature as a function of the position of the Fermi level. For the ice-X of H_2_O at 150 GPa we compute, within this procedure, an astonishingly high phononic superconducting coupling, leading to room-temperature superconductivity already at a doping of a few percent! Although widely used in the literature, we can not expect that such a rigid shift of the Fermi level yields more than an estimate for the order of magnitude of the critical temperature upon doping. In fact, the extreme electron-phonon coupling obtained by the rigid shift would induce a strong electronic response, leading to a complete breakdown of the rigid shift approximation. Moreover, this method does not account for important physical effects stemming from the metallic part of the electronic screening, such as the mechanism responsible for Kohn anomalies^90^ that can significantly modify the spectrum of phonons.

A more realistic way to study theoretically the effect of doping on the superconducting properties is to calculate the phonon and electron-phonon matrix elements. The phonon spectra and the electron-phonon matrix elements were obtained employing density-functional perturbation theory^91, 92^, as implemented in the plane-wave based code abinit^93^.

**Figure 6:**
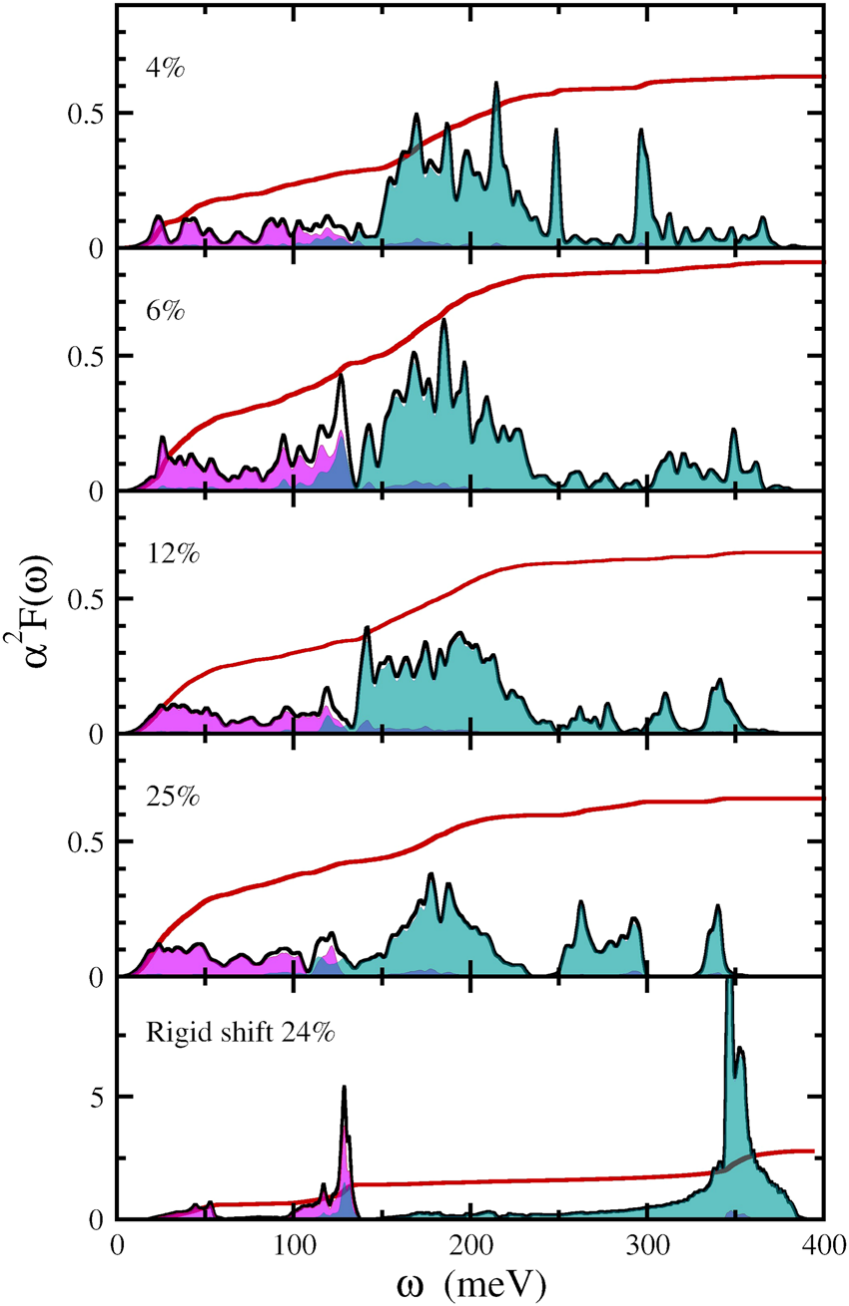
Eliashberg spectral function (black lines) and integration curve of the electron-phonon coupling constant *λ*(*ω*) (red lines) for hole-doped H_2_O in its phase X at 150 GPa. Doping level is indicated in each panel. Shaded areas are the projections on atomic displacements of oxygen/nitrogen (violet) and hydrogen (light blue).

**Figure 7:**
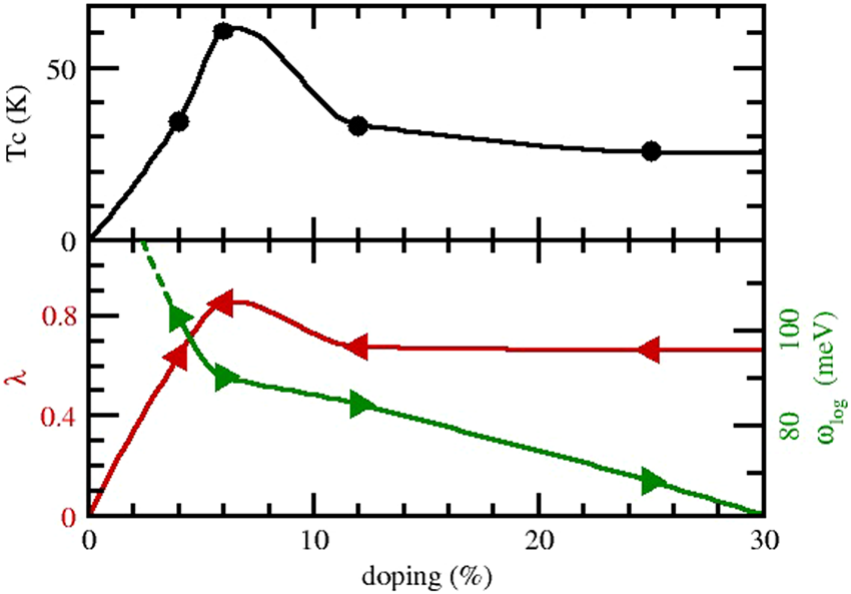
Top panel: calculated critical temperatures with the McMillan-Allen-Dynes formula as a function of doping of H_2_O in its phase X at 150 GPa. Lower panel: electron phonon coupling constant *λ* (red left triangles and left axis) and average phonon frequency *ω*_log_ (green right triangles and right axis). Solid lines are a guide to the eye.

As we have seen, doping turns out to have a dramatic effect on the electronic structure of ice, in the phonon spectrum (not shown) and in the Eliashberg spectral functions, shown in Fig. 6 as a function of doping at 150 GPa. Comparing the supercell calculations with results obtained with a rigidly shifted Fermi level (see Fig. 6), we observe a complete restructuring of the phonon energies and coupling strength. The metallization provides a significant screening causing both a softening of the phonon frequencies and a reduction of the deformation potential. Nevertheless we still observe a significant electron-phonon coupling, as can be seen from the Eliashberg functions as well as from the logarithmic average of the phonon frequency *ω*_log_^89, 94^ (see Fig. 7). We furthermore calculated the phonon band-structure for B, C, N, and P doping. We find that all systems are highly unstable with large imaginary frequencies, with the only exception being nitrogen, that leads to dynamically stable structures in the doping range studied.

For low doping (<12.5%) two major contributions to *λ* can be distinguished: (i) the low frequency optical phonons (oxygen vibrations) that couple with 2*p* nitrogen states; and (ii) the mid-frequency range 150–200 meV (1100–1800 cm^−1^) that couples with the covalent oxygen-nitrogen hybridized state. For 25% doping all phonon branches contribute significantly to the *e* − *p* coupling, since at this doping the structure adopts a completely metallic character.

From these parameters we can easily estimate the critical temperature by means of the McMillan-Allen-Dynes^95^ formula. This gives T_C_ in the range from 20 to 60 K (assuming μ* = 0.1), with the maximum value reached for a doping level of 6.25% (see Fig. 7). Although lower than the astonishing value found in sulphur hydride (200 K) and considerably lower than the rigid shift prediction (300 K) this is still a sizable value, much larger than the T_C_ $\lesssim$ 4 K found in doped semiconductors at ambient pressure.

## Discussion

We now turn to the question of how one could synthesize the doped phase of ice under pressure. One possible path is to use a high pressure synthesis similar to the one used to obtain H_2_+H_2_O clathrates^71^. This involves inserting, at room temperature, H_2_ molecules inside the H_2_O crystalline *C*_1_ (clathrate) phase of water at 0.7 GPa. Experimentally, the unit cell of the *C*_1_ phase contains 36 water molecules in a channel-like arrangement, which can host up to six hydrogen molecules^96, 97^. One could start the synthesis with the analogous N_2_+H_2_O clathrate (*C*_1_), where the percentage of filled N_2_ molecules will determine the doping level at high pressure^98^. However, it is well-known that N_2_ is one of the most stable molecules in the universe. Therefore, following this path would require high energies to break the strong N–N covalent bond, achievable perhaps only with strong laser heating.

Another possibility is to start with a less stable molecule, such as ammonia (NH_3_). The stability of ammonia is greatly reduced under pressure, and indeed it has been reported that it forms super-ionic phases^99^. These compositions have been extensively studied and well documented, however, only up to relatively low pressures of 50 GPa^100^.

A third possibility is to start the synthesis with admixtures of other molecules, such as nitric oxide (NO), nitrogen dioxide (NO_2_) or azanide H_2_N to name a few. Unfortunately, all of these mixtures have been scarcely studied at high pressure.

In conclusion, we investigated the possibility of inducing high-temperature superconductivity by doping insulating hydrides under pressure. By taking the phase X of ice as an example, we studied how the phonon spectra and the electron-phonon coupling evolve as a function of doping. Several dopants were analyzed, with the conclusion that only nitrogen leads to dynamically stable structures that are hole-doped. It turns out that for rather reasonable values of doping, one can reach superconducting transition temperatures as high as 60 K at 150 GPa. Considering the vast number of hydrides that remain insulating under pressure and that can be doped, this result opens a number of possibilities for the exploration of high-temperature superconductivity in these systems.

## Methods

The energies, atomic forces and stresses were evaluated within density functional theory with the Perdew-Burke-Erzernhof (PBE)^101^ approximation to the exchange-correlation functional. A plane wave basis-set with a high cutoff energy of 1000 eV was used to expand the wave-functions together with the projector augmented wave (PAW) method as implemented in the Vienna Ab Initio Simulation Package vasp^102^. Geometry relaxations were performed with tight convergence criteria such that the forces on the atoms were less than 2 meV/Å and the stresses were less than 0.1 meV/Å^3^.

The reference structures for hydrogen are *P*6_3_ *m* (0–120 GPa) and *C*2/*c* (120–300 GPa) from ref. 45. The reference phase for oxygen is *C*2/*m*, *ζ* structure^103–105^.

The phonon spectra and the electron-phonon matrix elements were obtained with density-functional perturbation theory^91, 92^, as implemented in the plane-wave based code abinit^93^. For the electron-phonon the following *k* and *q*-meshes were used for the different supercells: 25% doping, *k* = 16 × 16 × 16, and *q* = 8 × 8 × 8; 12.5% doping, *k* = 8 × 8 × 8 and *q* = 4 × 4 × 4; 6.25% doping, *k* = 4 × 4 × 4 and *q* = 4 × 4 × 4; 4.16% doping, *k* = 2 × 2 × 2 and *q* = 2 × 2 × 2.

For defect formation energy calculations we used *k* = 2 × 2 × 2. Formation energies for oxygen (*V*_O_) and hydrogen (*V*_H_) vacancies were calculated using the standard formulation.

1 $${E}^{f}={E}_{D}^{q}-{E}_{{{\rm{H}}}_{2}{\rm{O}}}-\sum _{i}\,{n}_{i}[{\mu }_{i}+{\rm{\Delta }}{\mu }_{i}]+q[{\varepsilon }_{{\rm{VBM}}}+{\rm{\Delta }}{\varepsilon }_{F}]+{E}_{{\rm{cor}}}$$

where ${E}_{D}^{q}$ is the energy of a supercell, here 3 × 3 × 3, containing a vacancy *D* in a charge state *q*, ${E}_{{{\rm{H}}}_{2}{\rm{O}}}$ is the energy of the same size pure crystal supercell, *n*_*i*_ is the number of elements added to the supercell and *μ*_*i*_ is the chemical potential of an element *i* defined as the energy per atom of the reference phase (for H_2_, N_2_ and O_2_ the stable phases at 150 GPa were used). The quantity Δ*μ*_*i*_ is dependent on the experimental conditions, but can be viewed as a free parameter bound by the stability of the crystal ice phase. Finally, *ε*_VBM_ is the energy of the valence band minimum (VBM) of the pure crystal, Δ*ε*_*F*_ is the value of the Fermi level referenced to the VBM, and *E*_cor_ is the electrostatic correction energy, including an alignment term, calculated using the SXDEFECTALIGN code^106^. All formation energy calculations were performed including local atomic environment relaxations only. Formation enthalpies were obtained using Eq. 2, where ${E}_{{\rm{relax}}}^{f}$ is calculated via Eq. 1 with volume relaxations in the calculation of supercell energies.

2 $${H}^{f}={E}_{{\rm{relax}}}^{f}+P{V}^{f}$$

The dielectric constant used for electrostatic corrections included both the static dielectric and the ionic contributions. We did not considered changes in the electrostatic energy with the relaxation volume.

## Electronic supplementary material

Supplementary Information
