# Supplementary material for: Emergence of superconductivity in doped H2O ice at high pressure
Source: Sci Rep. 2017 Jul 28;7:6825. doi: 10.1038/s41598-017-07145-4 (PMC5533783; doi:10.1038/s41598-017-07145-4)
Supplement: Supplementary file 1 — Supplementary Information [file 41598_2017_7145_MOESM1_ESM.pdf]

Supplemental Material for
Emergence of superconductivity in doped H_2O ice at high pressure

José A. Flores-Livas, Antonio Sanna, Miglė Grauzinyte, Arkadiy Davydov, Stefan Goedecker and Miguel A. L. Marques

May 12, 2017

1 Hole doped structure of ice at high pressure

In order to simulate an hole doped ice crystal we substitute oxygen for nitrogen in ratios of 25%, 12.5%, 6.25% and 4.16% in the simulation supercells show in Fig. 1. Structural relaxation was then carried out for the supercells at 150 GPa. Low doping (4–6%), induces fairly small modifications to the ice-X crystal structure, while larger doping levels have a stronger effect on the local environment, as reflected in the volume expansion. Nevertheless the ice-X structural motif is preserved.

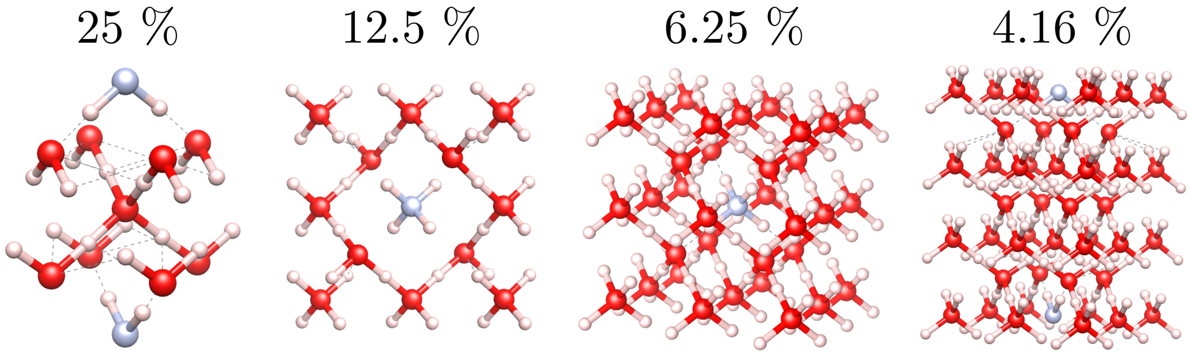

Figure 1: Simulation supercells used at different doping levels.

2 Crystal structure of N-doped ice-X

In Table 1 we give details of the supercell model of N-doped ice-X used for this work.

3 Stability and formation enthalpy of the H–O–N system

We studied the stability of the doped compositions with nitrogen by means of total energy DFT calculations. The region of the ternary phase diagram (H–O–N) studied is zoomed in the top panels of Fig. 2. Solid blue dots represent the ground-state (experimentally know structures) elemental phases of H_2, O_2 and N_2 occurring at 150 GPa. As expected, we find that the phase X of ice is the stable composition in this region of the H-O-N phase diagram (green circles). In the two bottom panels we plot the stability

Table 1: Crystal structure parameters for the doped supercells of the phase X of ice (relaxations at 150 GPa).

Composition (Pressure)	Crystal (spgrp)	Lattice parameters (Å)	Atom type	WP	Atom coordinates (internal coordinates)
$H_2O_{0.75}N_{0.25}$	$P42m$	$a = 2.52145$	H	$4n$	0.24756 0.24756 0.62448
(150 GPa)	(111)	$b = 2.52145$	H	$4n$	0.73386 0.73386 0.11316
		$c = 5.56810$	O	$2g$	0.00000 0.00000 0.73886
		$\alpha = 90.0$	O	$1b$	0.50000 0.50000 0.50000
		$\beta = 90.0$	N	$1d$	0.50000 0.50000 0.00000
		$\gamma = 90.0$			
$H_2O_{0.875}N_{0.125}$	$P42m$	$a = 5.25065$	H	$8o$	0.87574 0.37395 0.74495
(150 GPa)	(111)	$b = 5.25065$	H	$4n$	0.61458 0.61458 0.73580
		$c = 2.54502$	H	$4n$	0.87426 0.87426 0.75826
		$\alpha = 90.0$	O	$4n$	0.75602 0.75602 0.01405
		$\beta = 90.0$	O	$2f$	0.50000 0.00000 0.50000
		$\gamma = 90.0$	O	$1c$	0.00000 0.00000 0.50000
			N	$1b$	0.50000 0.50000 0.50000
$H_2O_{0.9375}N_{0.0625}$	$P43m$	$a = 5.18627$	H	$12i$	0.37602 0.37602 0.12528
(150 GPa)	(215)	$b = 5.18627$	H	$4e$	0.12670 0.12670 0.12670
		$c = 5.18627$	H	$12i$	0.12407 0.12407 0.62727
		$\alpha = 90.0$	H	$4e$	0.61579 0.61579 0.61579
		$\beta = 90.0$	O	$4e$	0.25038 0.25038 0.25038
		$\gamma = 90.0$	O	$3c$	0.00000 0.50000 0.50000
			O	$4e$	0.75618 0.75618 0.75618
			O	$3d$	0.50000 0.00000 0.00000
			O	$1a$	0.00000 0.00000 0.00000
			N	$1b$	0.50000 0.50000 0.50000
$H_2O_{0.9583}N_{0.0416}$	$P42m$	$a = 5.17922$	H	$8o$	0.87522 0.36997 0.74963
(150 GPa)	(111)	$b = 5.17922$	H	$4n$	0.61393 0.61393 0.07818
		$c = 7.78908$	H	$4n$	0.87617 0.87617 0.08474
		$\alpha = 90.0$	H	$8o$	0.87473 0.37526 0.08288
		$\beta = 90.0$	H	$4n$	0.37527 0.37527 0.41564
		$\gamma = 90.0$	H	$8o$	0.87591 0.37826 0.41577
			H	$4n$	0.87198 0.87198 0.41991
			H	$4n$	0.37459 0.37459 0.75185
			H	$4n$	0.87614 0.87614 0.74795
			O	$4n$	0.75689 0.75689 0.17137
			O	$4m$	0.00000 0.50000 0.66554
			O	$4n$	0.75142 0.75142 0.83303
			O	$4n$	0.25202 0.25202 0.50031
			O	$2e$	0.50000 0.00000 0.00000
			O	$1a$	0.00000 0.00000 0.00000
			O	$2h$	0.50000 0.50000 0.33125
			O	$2g$	0.00000 0.00000 0.33678
			N	$1d$	0.50000 0.50000 0.00000

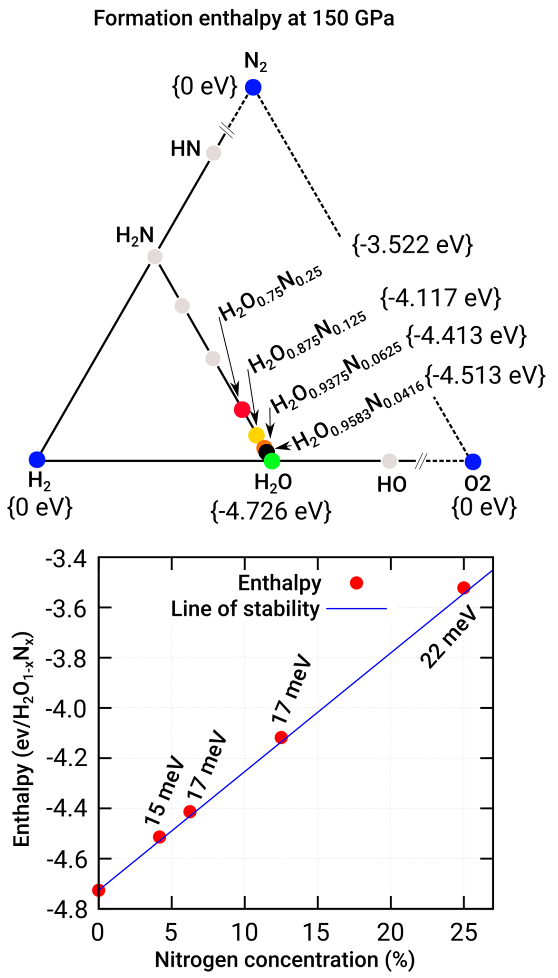

Figure 2: Left panel shows the formation enthalpy for the doped structures at 150 GPa. Right panel shows the enthalpy including zero point energy motion. Bottom panels represent the line of stability between H_2O and an hypothetical H_2N composition.

line between H_2O to an hypothetical H_2N composition respectively without (left) and with (right) zero point energy corrections (ZPE). Without ZPE all doped structures (4.16%, 6.25%, 12.5%, and 25%) lie only ∼15 meV per formula unit above the stability line. The structures are further stabilized by the ZPE: for low doping (4% and 6%) the structures are a mere 2 meV per formula unit above the stability line (metastable compositions); while for 12.5% and 25% we find the doped structures well below the stability line.

4 Defect formation enthalpies

At high pressures the relevant quantity for the formation of defects is the formation enthalpy H^f, rather than the formation energy E^f, unfortunately the additional PV^f term is not straightforward to evaluate, as the definition for the formation volume V^f of a defect is not properly defined for non-elemental systems. In the article we attempt to highlight the effect of the neglected PV^f term by following a chemical potential analogy to evaluate the volume of formation for defects in H_2O.

In an elemental solid the formation energy of a vacancy ($N' = N - 1$) or interstitial ($N' = N + 1$)

Defect	Charge state	V^{rel}	V^f
V_O	q=-2	5.66	10.88
V_O	q=-1	0.71	5.93
V_O	q= 0	-3.77	1.45
V_O	q= 1	-7.57	-2.35
V_O	q= 2	-9.29	-4.07
V_H	q=-1	2.79	4.51
V_H	q= 0	-1.3	0.42
V_H	q= 1	-5.36	-3.64
N_O	q=-1	4.89	5.08
N_O	q= 0	1.05	1.24
N_O	q= 1	-2.71	-2.52

Table 2: Calculated formation volumes of defects in ice-X

can be defined as Eq. 1, where ϵ is the energy per particle in the reference system, i.e. $\epsilon = \frac{E(N)}{N}$.

$$E^f = E(N') - \frac{N'}{N}E(N) = [E(N') - E(N)] - [N' - N]\epsilon \tag{1}$$

For compounds or substitutional species, the second term in the right hand side of Eq. 1 is often replaced by a chemical potential term $[N^* - N]\epsilon \to n_i\mu_i$. This is equivalent to saying that the atoms n_i removed (added) from the cell did not simply disappear (appear), but were rather put in a different environment with energy μ_i. It is convenient to rewrite the chemical potential of species i in two terms $\mu_i = \mu_i^0 + \Delta\mu_i$, where $\mu_i^0 = \epsilon_i$, to highlight the fact that there is some flexibility in the chemical potentials of individual species as long as phase-stability conditions are respected. In the case of H_2O, for example, $\Delta\mu_{\mathrm{O}} + 2\Delta\mu_{\mathrm{H}} = \Delta H^f(\mathrm{H_2O})$. The choice of which element takes up more of the contribution is simply representative of different experimental conditions.

We extend this idea also to the formation volumes, that are defined for elemental solids in Eq. 2, where v is the volume per atom in the reference system $v = \frac{V(N)}{N}$. The first term on the right-hand side of Eq. 2 is the relaxation volume, i.e. the total volume change in between the supercell with and without the defect.

$$V^f = V(N^*) - \frac{N^*}{N}V(N) = [V(N^*) - V(N)] - [N^* - N]v \tag{2}$$

We can then again select a reference system into which we will be putting our newly removed (added) atom $[N^* - N]v \to n_i v_i^*$, where by analogy $v_i^* = v_i^0 + \Delta v_i$, with v_i^0 straightforwardly taken as the volume per atom in the same reference phase used for the chemical potential. Unlike the chemical potential, however, $\Delta v_{\mathrm{O}} + 2\Delta v_{\mathrm{H}} = \Delta v^f(\mathrm{H_2O})$ condition does not have to be satisfied. In the case of a pure H_2 environment for example $\Delta v_{\mathrm{H}} = 0$, but what then is an appropriate choice for Δv_{O}?

All calculations that do obey the volume conservation $\Delta v_{\mathrm{O}}+2\Delta v_{\mathrm{H}} = \Delta v^f(\mathrm{H_2O})$ condition, correspond to the situation where the removed atoms are forming new layers of H_2O on the surface. For our calculations of formation enthalpies we chose to set $\Delta v_{\mathrm{O}} = \Delta v_{\mathrm{H}} = \frac{1}{3}\Delta v^f(\mathrm{H_2O})$, so that we do not favor one defect over the other.

The calculated formation volumes are shown in Table 4.
